# A framework for modeling performers' beat-to-beat heart intervals using music features and Interpretation Maps

**DOI:** 10.3389/fpsyg.2024.1403599

**Published:** 2024-09-04

**Authors:** Mateusz Soliński, Courtney N. Reed, Elaine Chew

**Affiliations:** ^1^School of Biomedical Engineering and Imaging Sciences, Faculty of Life Sciences and Medicine, King's College London, London, United Kingdom; ^2^Engineering Department, Faculty of Natural, Mathematical and Engineering Sciences, King's College London, London, United Kingdom

**Keywords:** RR intervals, heart rate variability, cardiac modeling, music performance, Interpretation Map, music features

## Abstract

**Objective:**

Music strongly modulates our autonomic nervous system. This modulation is evident in musicians' beat-to-beat heart (RR) intervals, a marker of heart rate variability (HRV), and can be related to music features and structures. We present a novel approach to modeling musicians' RR interval variations, analyzing detailed components within a music piece to extract continuous music features and annotations of musicians' performance decisions.

**Methods:**

A professional ensemble (violinist, cellist, and pianist) performs Schubert's Trio No. 2, Op. 100, Andante con moto nine times during rehearsals. RR interval series are collected from each musician using wireless ECG sensors. Linear mixed models are used to predict their RR intervals based on music features (tempo, loudness, note density), interpretive choices (Interpretation Map), and a starting factor.

**Results:**

The models explain approximately half of the variability of the RR interval series for all musicians, with R-squared = 0.606 (violinist), 0.494 (cellist), and 0.540 (pianist). The features with the strongest predictive values were loudness, climax, moment of concern, and starting factor.

**Conclusions:**

The method revealed the relative effects of different music features on autonomic response. For the first time, we show a strong link between an interpretation map and RR interval changes. Modeling autonomic response to music stimuli is important for developing medical and non-medical interventions. Our models can serve as a framework for estimating performers' physiological reactions using only music information that could also apply to listeners.

## 1 Introduction

Live music provides a unique context to study cardiac and other physiological responses in ecological and engaging settings. For both listeners and musicians, music is an auditory and mental stimulus affecting the rest of the body through the autonomic nervous system (Grewe et al., 2007; Purwins et al., 2008; Ellis and Thayer, 2010). Music elicits emotions (Yang et al., 2021) and strongly modulates autonomic responses (Labbé et al., 2007), which can be measured through physiological reactions; for example, heart and respiratory rate changes (Bernardi, 2005; Bernardi et al., 2009; Hilz et al., 2014), goosebumps, shivers, and chills (Grewe et al., 2007).

However, musicians must engage in physical activity and mental coordination while performing. Compared to music listening, performing modulates autonomic response more strongly (Nakahara et al., 2011). One of the most relevant measures of physiological response to music stimuli is the time distances between R-peaks, the most obvious part of the electrocardiograph (ECG) signal, called RR intervals, which correspond to depolarization and contraction of the ventricles. The length of the intervals and their variation in time are modulated by the autonomic nervous system (ANS), which consists of two branches: parasympathetic, which increases RR intervals (decreases heart rate) and increases total heart rate variability (HRV), and sympathetic, which does the inverse (Sloan et al., 1994). HRV is indicative of the body's ability to cope with mental and physical stress and environmental factors (Nishime, 2000; Pierpont et al., 2000; Pierpont and Voth, 2004; Cammarota and Curione, 2011; Michael et al., 2017; Pokhachevsky and Lapkin, 2017). Healthy individuals have higher HRV, while lower variability can indicate poor sympathovagal balance and resultant cardiovascular disease (van Ravenswaaij-Arts, 1993).

Studies on how music affects the ANS have been performed mostly on listeners' heart rate (HR), HRV, and cardio-respiratory functions due to music-related arousal and relaxation (Chlan, 2000; Bernardi, 2005; Bernardi et al., 2009; Bringman et al., 2009; Nomura et al., 2013; Hilz et al., 2014). Existing studies examined music features like music genre (Bernardi, 2005; Hilz et al., 2014), the complexity of the rhythm (Bernardi, 2005), and tempo (Bernardi, 2005; Nomura et al., 2013), and music structures such as vocal and orchestral crescendos, and musical phrases and emphasis (Bernardi et al., 2009).

On the other hand, studies of musicians' autonomic response have focused mainly on stress and the effect of the performance context rather than the impact of the music itself. Prior studies considered changes in mean physiological signal parameters with factors such as ecological setting—rehearsal vs. public performances of pieces by Strauss, Mozart, Rachmaninov, and Tchaikovsky by the BBC Orchestra (Mulcahy et al., 1990); a selection of easy and strenuous classical pieces (Harmat and Theorell, 2009), auditioning with and without audience playing Bach's *Allemandes* (BWV1004 or BWV1013; Chanwimalueang et al., 2017), onstage and offstage stress of the opera singers performing Mieczys Weinberg's *The Passenger* (Cui et al., 2021)—piece difficulty (Mulcahy et al., 1990; Harmat and Theorell, 2009), music genre—classic rock, hard rock, Western Contemporary Christian (Vellers et al., 2015)—musicians' flow states (Horwitz et al., 2021), and intensity of physical effort on different instruments as measured by a maximum theoretical heart rate (Iñesta et al., 2008). Musical action goes beyond physical effort. Hatten describes "effort" in music with respect to planning and action required to overcome environmental and physical forces to achieve musical intention (Hatten, 2017). Musicians must maintain careful interplay between their intention, effort, and restraint to control their technique, stay within the bounds of their instruments and bodies, and execute their intended articulation and expression (Tanaka, 2015). Considering the time frame of performance, music affects musicians in all three time domains: the present (the currently played music), the past (recovering from previous playing), and the future (anticipating the next action).

There is a lack of work analyzing musicians' autonomic responses in relation to detailed components of the music piece on a continuous scale rather than taking average measurements under different physical conditions and stress levels. An example of analysis in the continuous scale was shown in the study by Williamon et al., which examined RR intervals and HRV parameters collected from a pianist (Williamon et al., 2013). It analyzed the stress level while performing Bach's English Suite in A minor (BWV 807) for a large audience compared with one in the lab using defined stress markers: mean RR intervals, HRV frequency parameters, and sample entropy. The results suggested that autonomic responses are more powerful in live, ecological settings. The analysis also combined the basic performer's annotations of challenge during play (the first and third movements were marked as the most challenging) and changes in stress levels as measured physiologically.

In this study, we bridge this gap by examining three collaborating musicians (violin, cello, and piano) playing Schubert's Trio No. 2, Op. 100, andante con moto, measuring their ECG signals and predicting their RR intervals based on continuously measured music features (loudness, tempo, and note density) and musicians' annotations serving as a trace of their interpretation of the piece. The features annotated were selected as potentially important to physiological response during performance. Loudness and tempo have previously been found as independent factors that can elicit emotional states (valence/arousal) or cardiac response in listeners (Bernardi, 2005; Yang et al., 2021). It is expected that they may also be important in the autonomic response of musicians. Note density was selected as an indirect measure of the effort associated with playing intensity.

We selected RR intervals—the inverse of the heart rate—as the continuous measure of physiological response to playing music. This approach allowed us to model the instantaneous reactions to specific music structures and to changes in the music signals. We opted not to use HRV measures because they require a moving window from 10 s to a few minutes long, depending on the HRV parameter, which can be problematic when analyzing relatively short pieces, some of which may be only 2–3 min long, as was the case in our dataset. While in ultra-short HRV analysis, the smallest time window can be as short as 10 s (e.g., for RMSSD during cognitive task, Salahuddin et al., 2007), the values calculated in this way are highly sensitive to outliers, producing unsatisfactory results, especially for RR time intervals during music playing which are far from stationary. Hence, we chose to focus on RR intervals which allows our framework to be applied to shorter and highly variable music pieces.

We present the framework for modeling physiological reactions using regression mixed models that take as input a novel representation, which we call the Interpretation Map. We hypothesize that such maps can provide step improvement to models of individual beat-to-beat heart intervals. This is the first time, to our knowledge, that such a method has been used to describe beat-to-beat changes of RR intervals in musicians. Such information about music-derived physiological responses could be used to inform future training and therapeutic applications.

## 2 Methods

### 2.1 Participants

The study participants were a trio of professional musicians (over 20 years of performance experience, more than 10 years playing together). We asked the musicians to get at least 7 h of sleep, avoid caffeinated and alcoholic beverages for 8 h, and eat for 1 h prior to the agreed rehearsal time.

### 2.2 Music selection

The trio performed Schubert's Trio No. 2, Op. 100, Andante con moto (henceforth, "the Schubert"). The piece offered a balance between varied musical features and clear musical structures. The alternating sections marked by the musical themes enabled comparisons between similar and repeated musical features. The tempo of the piece (about 88 BPM) resembles a typical human HR, which allows us to align musical features to physiological data without over sampling the signals. The musicians had not practiced the Schubert together before the first recording.

### 2.3 Recording equipment

We use the Polar H10 (Polar Electro Oy, Kempele, Finland) heart rate sensor to measure the ECG signal in each performance. The Polar unit registers a one-channel ECG signal with a sampling frequency of 130 Hz, but it detects QRS complexes to create RR interval series at a higher sampling frequency of 1000 Hz. Data samples from the Polar straps are collected via Bluetooth on three iPhones (Apple, Cupertino, CA, USA) running the HeartFM mobile app on iOS 16.1.2 or higher. We use a Zoom H5 (Zoom, Tokyo, Japan) handheld recorder for audio recording.

### 2.4 Procedure

**Figure 1:**
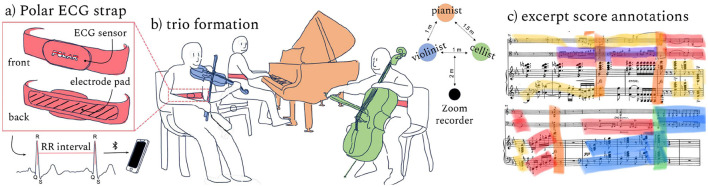
Data collection: the trio perform the Schubert nine times while wearing Polar straps **(a)** to collect ECG data. A Zoom recorder gathers audio data from each performance **(b)**. Players annotate categories of performed structures in the music score **(c)**.

The Polar straps (Figure 1) are moistened and worn across each musician's sternum. The musicians are seated in a typical trio configuration. The Zoom recorder is placed ~2 m from the musicians. The three iOS devices and Zoom are synchronized with a clapper for later alignment of the signals. The Schubert is recorded nine times over 5 different days. The musicians performed the piece as written, after which the data recordings were stopped. The Schubert was rehearsed and recorded nine times over 5 different days.

### 2.5 Score annotation

On a separate date following the recordings, the musicians collaborate to annotate the music score on the right side of Figure 1, developing a novel set of categories reflecting their negotiated roles and collective actions. We refer to this representation as the Interpretation Map. The Interpretation Map captures the musicians' experienced cognitive and physical load, expressive choices, and how they negotiated a path through the music. The trace of the musicians' intention, immersion and reflection embedded in this Interpretation Map represent key components of the performances that likely affected their heart rates. The Interpretation Map consisted of seven salient categories of musical action: (1) melodic interest (main melody), (2) melodic interaction (e.g., melody and counter-melody together), (3) dialogue (e.g., asynchronous call and answer), (4) significant accompaniment (as opposed to accompaniment that is background), (5) climax (usually preceded by a build-up into the climax, like a crescendo), (6) return / repose (however, it was not used in the model due to low occurrence), and (7) moment of concern (during which musicians need to manage greater risk). These particular "moments of concern" were moments that were persistently concerning across repetitions. Thus, the musicians needed to manage greater risk at these parts of the piece across performance numbers.

The final annotated score, agreed upon collaboratively by the trio, reflects the role of each instrument in the piece, with the piano supplying the accompaniment more often than the cello or violin. In working with the trio, we presumed a level of musical expertise in the study, namely, music theory knowledge, the ability to read and interpret musical scores and markings (e.g., dynamics, bowings, and articulations), harmonic awareness, focus and concentration, technical proficiency on respective instruments (e.g., control of tempo, pitch, and rhythm), and proficiency in performing with others in a music ensemble. The annotations made by the musicians in this study depend on their collective musical expertise and awareness of the music genre. They are not representative of all possible interpretations by other musicians but are relevant to this particular trio and the performances examined here.

### 2.6 Data preparation

#### 2.6.1 Score-time domain

To focus on musically salient features and their effect on physiological data across multiple performances, we synchronize the RR interval time series and performance audio to score-time (Chew and Callender, 2013)—i.e., with musical beats instead of seconds as time axis—to match them to the performers' score annotations. Other continuous time signals were adjusted to the annotated beats using linear interpolation. The conversion to score-time also matches the physiological signals to music features extracted from the score and makes the signals from all performances comparable. We use the half-beat and the eighth note pulse in the 2/4 m as a unit for the 848 eighth note beats in Schubert.

#### 2.6.2 Physiological data

The RR interval series generated by Polar's automatic QRS complex detection are reviewed for inaccurate beat detection and premature beats; premature atrial and ventricular beats are removed. In each performance, the series of normal beats were normalized to the performer separately. The normalization of the RR intervals allowed us to compare the effect of the music structures annotated in the Interpretation Map between the musicians. Since the musical parameters extracted from the score are common for each performance, we wanted to assess their effects independent of the baseline RR interval value.

#### 2.6.3 Music features

We compute three music features related to the musicians' physical effort while playing: note density, loudness, and tempo. Loudness and tempo signals were calculated based on the recordings of the trio's combined performance. We decided to process the full recording instead of recording each musician playing separately to capture the real-life situation of the musicians playing together as an ensemble.

We manually annotate the eighth note pulse in the recorded performance audio using Sonic Visualizer (Cannam et al., 2010); the annotations are corrected to note onsets using the TapSnap algorithm of the CHARM Mazurka Project^1^. We calculate note density, the number of note events per beat, using the *notedensity* function in the Matlab MIDI Toolbox (Eerola and Toiviainen, 2004; Toiviainen and Eerola, 2016) as a measure of technical complexity. This time series was calculated separately for each musician based on the score. Perceptual loudness in sones was derived using the Music Analysis Matlab Toolbox (Pampalk, 2004). The tempo was computed from the beat annotations in beats per minute (BPM). Tempo and loudness from each performance are normalized, and loudness is filtered with a low-pass Butterworth filter with order *N* = 3 and *W*_*n*_ = 0.125 (cutoff frequency parameter in butter function in Python).

#### 2.6.4 Score annotations

The musicians' score annotations are transformed into signal inputs for the models. A length *L* binary vector (where *L* = 848, the number of eighth note samples) is created for each annotated category, with 1 indicating the occurrence of an annotation (0 otherwise). From these binary vectors, we generate the reference for the input signals, which are the sum of Gaussian kernel density estimation functions (Silverman, 2018). When the *i*-th element of the binary vector is 1, a Gaussian function (standardized to sum = 1) centered at index *i*+16th (four bars) with SD = 16/2 (two bars) is added to the final vector. These parameters relate to the 2/4 m of the Schubert.

#### 2.6.5 Starting factor

The musicians' physiological responses are observed to be different at the beginning of playing due to individual activation of autonomic mechanisms underlying cardiovascular reactivity rather than any specific physical or musical features. The first part of Schubert is not physically challenging (low loudness, simple piano accompaniment, and calm introduction of the cello theme); the decline in RR intervals is relatively pronounced through the first 20 bars of music. The initial stress reaction to mental tasks caused by the process of switching from rest to stimulation has been associated with the disruption of baseline homeostasis (Kelsey et al., 1999; Hughes et al., 2011, 2018; Widjaja et al., 2013). We model this initial physiological reaction by introducing a starting factor, tailored to the Schubert, expressed as a time series based on the formula:


(1) $$\{\begin{array}{ccc} t^{b_{1}*m(t)+b_{2}*a(t)} & \text{when} & t\in [0,80]\\ 0, & \text{when} & t>80, \end{array}$$

where *t* is the score-time, *m*(*t*) and *a*(*t*) terms are binary indicators of whether the musician plays a melody or accompaniment (or significant accompaniment) at score-time *t*, and *b*_1_ = −0.2364 and *b*_2_ = −0.0871, mean values of the exponents obtained by fitting *t*^*b*^ to each musician's first 80 RR intervals (in score-time) from the cellist (*b*_1_) and pianist (*b*_2_) over all performances. Thus, for the cellist, who plays the opening melody, the factor equals *t*^−0.2364^, and for the pianist, *t*^−0.0871^, where *t*∈[0, 80], and 0 otherwise. Note that the violin does not play during the first 20 bars, and her starting RR intervals were like the baseline; hence, the starting factor for the violinist has a rectangular shape and equals $t^{b_{1}*0+b_{2}*0}=1$.

### 2.7 Statistical analysis

Linear mixed models are used to predict the musicians' RR intervals. Three models with differing complexity are created for each musician:

**Model Set 1:** Considers only loudness and tempo extracted from the recorded audio files for the full ensemble.

**Model Set 2:** Includes tempo, loudness, and the factors calculated individually for each musician: note density and the Interpretation Map, which consists of annotations of melodic interest, dialogue, accompaniment, significant accompaniment, climax, and moments of concern.

**Model Set 3:** Includes tempo, loudness, note density, the Interpretation Map, and the starting factor time series.

The features in Model Sets 1–3 were considered as fixed effects. The performance number, 1–9, was added to the models as a random effect (random intercept). Thus, we progressively observe the model's performance as its complexity increases.

The model coefficients of the LME models are calculated using the bootstrapping method—sampling with replacement from the original dataset 1,000 times (Haukoos, 2005). In the bootstrapping method, the p-values are estimated by generating distributions for the coefficients with mean 0 using coefficient and mean values extracted from the original data, i.e., distributions for the null hypothesis that the feature has no effect in the model. Then, we calculate the probability, considering a two-tailed hypothesis, that the mean is significantly different from zero. If the probability is < 0.05, we reject the null hypothesis that the feature has no effect in the model. The backward stepwise method based on the Akaike Information Criterion (AIC) was used to eliminate insignificant variables in all models. The R^2^ (coefficient of determination) was used to estimate the variation in the dependent variable explained by the independent variables.

Additionally, the correlation coefficients between aggregated input variables from all performances—time series from audio signals, note density, the Gaussian Kernel Functions, and the Starting Factor—were calculated to check for strong pairwise associations. The variance inflation factor (VIF) and Cohen's *f*^2^ effect sizes were also computed for each variable and Model Set.

## 3 Results

**Figure 2:**
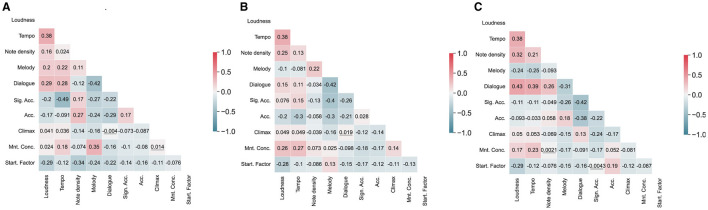
Correlation matrices for musical parameters calculated from audio signals (loudness and tempo), score (note density), and the Interpretation Map (Gaussian Kernel Functions) for each musician [**(A)** Violinist, **(B)** Cellist, and **(C)** Pianist]. Most of the correlations were significant (although small), and the non-significant correlation coefficients (*p* > 0.05) were underscored.

The results of the LMEs considering all independent variables (in Model Sets 1–3) for each musician are shown in Table 1. The correlation matrices for all musical parameters (loudness, tempo, note density, and the Gaussian Kernel Functions for categories from the Interpretation Map) are presented in Figure 2. **Although most coefficients were significantly different from zero (which may have been due to the relatively large sample size)**, we do not observe strong correlations (|*r*| < 0.50) between these parameters for any musicians' results. The VIF values are presented in Table 1. The VIF for all variables is between 1.13 and 5.17 (mean 2.04), which suggests low multicollinearity, except for the Violinist's Melody in Model Set 3, where VIF is larger than 5, suggesting high collinearity.

**Table 1:** Parameters used to model musicians' RR intervals whilst they performed the Schubert for the three model sets.

	**Violinist**			**Cellist**			**Pianist**		
**Model set 1**									
R^2^	**0.293**			**0.072**			**0.237**		
AIC	**−6, 054**			**−4, 475**			**−5, 104**		
	β	*f* ^2^	*VIF*	β	*f* ^2^	*VIF*	β	*f* ^2^	*VIF*
Intercept	$0.708_{0.686}^{0.732}$	−	−	$0.805_{0.783}^{0.827}$	−	−	$0.631_{0.607}^{0.653}$	−	−
Loudness	$-0.930_{-0.971}^{-0.891}$	0.243	1.17	$-0.359_{-0.399}^{-0.316}$	0.008	1.17	$-0.938_{-0.974}^{-0.899}$	0.229	1.17
Tempo	$-0.260_{-0.301}^{-0.221}$	0.038	1.16	$-0.309_{-0.347}^{-0.269}$	0.041	1.16	$-0.107_{-0.146}^{-0.071}$	0.007	1.16
**Model set 2**									
R^2^	**0.540**			**0.485**			**0.446**		
AIC	**−9, 255**			**−9, 092**			**−7, 592**		
	β	*f* ^2^	*VIF*	β	*f* ^2^	*VIF*	β	*f* ^2^	*VIF*
Intercept	$0.826_{0.803}^{0.848}$	−	−	$0.796_{0.773}^{0.818}$	−	−	$0.633_{0.605}^{0.658}$	−	−
Loudness	$-0.508_{-0.545}^{-0.475}$	0.104	1.40	$-0.176_{-0.217}^{-0.143}$	< 0.001	1.34	$-0.649_{-0.689}^{-0.611}$	0.126	1.46
Tempo	$-0.147_{-0.185}^{-0.108}$	0.014	1.61	$-0.159_{-0.190}^{-0.128}$	0.018	1.35	$0.126_{0.085}^{0.166}$	0.004	1.44
Note density	$-0.013_{-0.015}^{-0.011}$	0.024	1.24	$-0.008_{-0.009}^{-0.006}$	0.012	1.16	non-sign.	−	−
Melody	$-0.306_{-0.321}^{-0.291}$	0.305	2.52	$-0.137_{-0.153}^{-0.122}$	0.037	3.81	$-0.155_{-0.169}^{-0.141}$	0.058	2.00
Dialogue	$-0.293_{-0.308}^{-0.278}$	0.296	2.33	$-0.067_{-0.083}^{-0.051}$	0.010	3.09	$-0.318_{-0.337}^{-0.299}$	0.122	4.27
Sig. Acc.	$-0.361_{-0.391}^{-0.330}$	0.084	1.57	$0.048_{0.031}^{0.066}$	0.002	2.74	$-0.135_{-0.153}^{-0.115}$	0.025	3.38
Acc.	$-0.181_{-0.197}^{-0.165}$	0.076	1.56	$0.083_{0.067}^{0.101}$	0.009	2.10	$-0.101_{-0.114}^{-0.087}$	0.023	2.05
Climax	$-0.587_{-0.617}^{-0.559}$	0.264	1.13	$-0.636_{-0.661}^{-0.612}$	0.264	1.29	$-0.649_{-0.678}^{-0.623}$	0.213	1.43
Mnt. Conc.	$0.159_{0.124}^{0.196}$	0.007	1.19	$-0.435_{-0.465}^{-0.404}$	0.134	1.45	$-0.296_{-0.324}^{-0.266}$	0.047	1.67
**Model set 3**									
R^2^	**0.606**			**0.494**			**0.540**		
AIC	**−10, 489**			**−9, 308**			**−9, 205**		
	β	*f* ^2^	*VIF*	β	*f* ^2^	*VIF*	β	*f* ^2^	*VIF*
Intercept	$0.653_{0.628}^{0.677}$	−	−	$0.868_{0.850}^{0.886}$	−	−	$0.511_{0.488}^{0.537}$	−	−
Loudness	$-0.495_{-0.532}^{-0.460}$	0.118	1.40	$-0.246_{-0.278}^{-0.209}$	< 0.001	1.40	$-0.431_{-0.470}^{-0.393}$	0.078	1.54
Tempo	$-0.214_{-0.254}^{-0.174}$	0.029	1.62	$-0.143_{-0.178}^{-0.112}$	0.018	1.35	$0.094_{0.057}^{0.130}$	0.004	1.44
Note density	$-0.004_{-0.006}^{-0.002}$	0.003	1.32	$-0.008_{-0.010}^{-0.006}$	0.013	1.16	$0.0004_{-0.0007}^{0.0002}$	0.001	1.20
Melody	$-0.093_{-0.105}^{-0.082}$	0.015	5.17	$-0.195_{-0.206}^{-0.183}$	0.053	4.57	$-0.038_{-0.052}^{-0.023}$	0.003	2.41
Dialogue	$-0.083_{-0.095}^{-0.072}$	0.013	4.84	$-0.138_{-0.151}^{-0.125}$	0.023	3.98	$-0.228_{-0.247}^{-0.209}$	0.067	4.55
Sig. Acc.	$-0.052_{-0.083}^{-0.023}$	0.002	2.23	$-0.030_{-0.044}^{-0.016}$	< 0.001	3.53	$-0.027_{-0.046}^{-0.009}$	0.001	3.77
Acc.	non-sign.	−	−	non-sign.	−	−	$-0.093_{-0.108}^{-0.080}$	0.023	2.05
Climax	$-0.330_{-0.361}^{-0.299}$	0.070	1.55	$-0.712_{-0.735}^{-0.690}$	0.285	1.45	$-0.495_{-0.524}^{-0.469}$	0.136	1.53
Mnt. Conc.	$0.166_{0.132}^{0.204}$	0.008	1.19	$-0.506_{-0.536}^{-0.475}$	0.154	1.62	$-0.151_{-0.181}^{-0.124}$	0.012	1.79
Starting factor	$0.300_{0.284}^{0.315}$	0.167	3.01	$-0.156_{-0.179}^{-0.134}$	0.017	1.52	$0.327_{0.311}^{0.346}$	0.206	1.36

Included are coefficients (and confidence intervals) that are significant, 95%CI in upper and lower scripts, as well as *f*^2^ effect sizes and variance inflation factors (VIFs).

*p* < 0.01 for note density and significant accompaniment in the pianist's model (in Model Set 3); otherwise, *p* < 0.001.

The values of the R^2^ and AIC parameters for each model set and musician have been written in bold text.

Observe that the models with collective music audio features, the Interpretation Map, the individually calculated note density values, and the starting factor (Model Set 3) explained more than half of the variability of the musicians' RR interval time series for the violinist and the pianist and almost half for the cellist. Based on the R^2^ values, the greatest improvement in explaining the variability in the RR intervals is observed after adding the Interpretation Map (Model Set 2); adding the starting factor (Model Set 3) further boosted the results.

**Figure 3:**
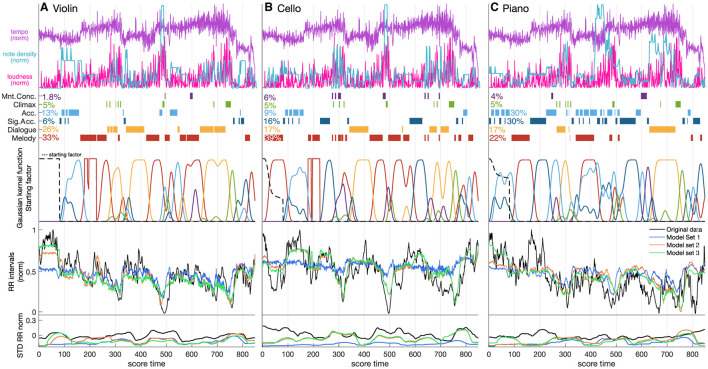
Distribution of tempo, loudness, Interpretation Map categories (melody, dialogue, significant accompaniment, accompaniment, climax, and moments of concern; together with their percentage occurrence in the piece for each musician), Kernel Gaussian Functions based on the aforementioned annotations, a time series introducing the starting factor (black dashed line), RR intervals, and standard deviation of RR intervals in score-time for each of the three musicians. The RR intervals are predicted using Model Sets 1 (tempo, loudness), 2 (+ Interpretation Map), and 3 (+ starting factor).

R^2^ increased with model complexity for all musicians and all groups of models (Model Sets 1–3). Model Set 1 (only audio parameters loudness and tempo) explained 29.3, 7.2, and 23.7% of the RR interval variability for the violinist, cellist, and pianist, respectively. Adding the Interpretation Map in Model Set 2 increased the R^2^ values to 54.0, 48.5, and 44.6%, respectively. The most complex Model Set 3, adding the starting factor, obtained the highest R^2^: 60.6, 49.4, and 54.0%, respectively. Figure 3 shows the original and predicted RR interval series from an example performance, together with the musicians' annotations and loudness and tempo signals in the score-time domain for all musicians. Observe that the reconstructed RR intervals from Model Set 3 follow the trends in the original data for all musicians. Their standard deviations also follow those in the original series, although the values are usually smaller.

We observe in Table 1 that most of the coefficients are negative, meaning that the musicians' RR intervals tend to decrease with the chosen features, but they vary in value. The results in the final Model Set 3 show that the components with the largest absolute values of weights are loudness (−0.495), climax (−0.330), and initialization factor (0.300) for the violinist; climax (−0.712), moment of concern (−0.506), and loudness (−0.246) for the cellist; climax (−0.495), loudness (−0.431), and initialization factor (0.327) for the pianist. Climax was the strongest common factor among all features for all musicians. We observe the largest simultaneous decrease in RR intervals at the beginning of climatic parts: from bar 67 (268 eighth notes in score time), from bar 115 (460 eighth notes in score time; the musicians' response in that part is a combined effect of climax, melodic interaction, and moments of concern) and from bar 158 (632 eighth notes in score time). A moment of concern is a type of stress. It has the lowest coefficient value for the cellist (−0.506). This is probably associated with a solo passage of 16th notes in bars 119–121 (476–484 eighth notes in into the piece). The coefficient of the moment of concern was slightly negative for the pianist (−0.151) but slightly positive for the violinist (0.166). However, the violinist labeled only one short segment of the piece (only three bars) as a moment of concern, which may be insufficient information to obtain a representative coefficient for this category. The rest of the annotations, including melody, dialogue, significant accompaniment, and accompaniment, have larger coefficient values (>−0.2), the singular exception being the pianist's annotation of the dialogue (−0.228), which for that instrument occurs largely with the climax.

Loudness and tempo have limited value for explaining the musicians' physiological response. Between the two, loudness has a stronger effect than tempo. Its coefficient has a larger absolute value for all musicians (based on the final Model Set 3): −0.495 (loudness) vs. −0.214 (tempo) for the violinist, −0.246 vs. −0.143 for the cellist, and −0.431 vs. 0.094 for the pianist.

Cohen's *f*^2^ effect sizes vary between musicians and Model Sets. In Model Set 1, the effect size for loudness is much higher than for tempo in the violinist's and pianist's models; in the cellist's model, *f*^2^ is small for both loudness and tempo. In Model Set 2, the effect size for loudness lessens due to the stronger influence of features from the Interpretation Maps: Melody (*f*^2^ = 0.305), Dialogue (*f*^2^ = 0.296) and Climax (*f*^2^ = 0.264) in the violinist's model; and, Climax in the cellist's (*f*^2^ = 0.264) and pianist's (*f*^2^ = 0.213) models. In Model Set 3, the effects of musical features in the models for violinists and pianists are masked by the Starting Factor, which models the RR intervals only at the beginning of the piece.

## 4 Discussion

The advantage of studying professional musicians is the ability to map actions onto a timeline based on high musical awareness and knowledge. The use of the Interpretation Maps in the models provided an insightful characterization of RR interval changes during music performance.

It is worth noting that the highest increase in *R*^2^ observed between Model Sets 1 and 2 is not only due to the fact that participant-specific predictors have been added but that these predictors capture the musicians' interpretative choices.

The music attains its greatest emphasis or most exciting part at the climax. These parts are characterized by a high degree of cohesion between the musicians. In a Time Delay Stability analysis of the current dataset (Soliński et al., 2023), climaxes were associated with the highest degree of physiological coupling between the musicians' RR intervals. The reaction to a musical climax can correspond to muscular exertion (Davidson, 2014) or emotional arousal (Bannister, 2018), although it may not be mentally or physically demanding. The highest heart rates (or lowest RR intervals) have been found during musical climaxes in previous studies on woodwind (clarinet) playing (Hahnengress and Böning, 2010).

The stronger effect of the loudness in comparison to tempo suggests that playing louder tends to decrease RR intervals more than playing faster for the Schubert. One exception is the positive tempo coefficient for the pianist, meaning the RR intervals increase with tempo, but the effect is relatively small. The positive effect of tempo on heart rate was previously reported as a significant factor in music playing (together with BMI and, to a lesser extent, instrument type) (Iñesta et al., 2008). We initially hypothesized that RR intervals would decrease with the number of notes per bar due to an increasing rate of movement; however, note density contributed only marginally to the model for all musicians.

Introducing the starting factor further improved model performance. The starting factor captures the initial disruption of homeostasis toward activation of the sympathetic nervous system. The music-based features in Model Sets 1 and 2 insufficiently accounted for the sharp decrease in RR intervals at the start of playing – compare the results for the different models in Figure 3 during the first 80 samples. This is likely due to the music features not encoding information that could predict such changes: there were no large changes in loudness and tempo, and no climax or moment of concern. The starting factor also depends on the musician's role during the initial bars. The largest reaction was observed for the cellist, who plays the main melody with piano accompaniment. Although the opening motif in the Schubert is relatively simple and easy to play, we observed a disproportionate drop in RR intervals at the start for the cellist, potentially boosted by the activation of the sympathetic nervous system. A slower decrease in RR intervals is observed for the pianist, who plays an accompanying part. The initialization factor for the violinist is constant, due to the silence during the first 80 eighth notes.

Simplicity is a strength of the models demonstrated here. They use only music-based information to model RR intervals. Another advantage is their explanatory value by evaluating the impact of specific factors on RR. Future models can include other physiological measures such as respiration, skin conductance, and physical movement. Estimating cardiac response to music playing using only information from the recorded performance—how musicians modulate musical expression—and interpretation—the musicians' decisions and actions—provides a strong link between the autonomic nervous system and music playing. This connection demonstrates the potential and forms a basis for using active music making in cardiovascular therapies.

## 5 Limitations

The model development has potential limitations. The results obtained are specific to the particular trio involved in this study, the selected music, and their current approach to performing the piece. The data will, and the results may, be different for another trio, another piece, and another period in their evolution as musicians. The starting factor requires tailoring to each piece based on music structures at the beginning of the piece and over time. The tailoring of the starting factor could be omitted by predicting only the RR intervals after the initial autonomic reaction. Note also that some pairwise associations (Figure 2) and collinearity measures (Table 1) suggest that caution should be exercised in interpreting the contributions of individual factors.

We used only one piece of Western Classical music to assess the impact of music features and performance decisions on musicians' RR intervals. Performing other pieces are expected to require different interpretation approaches and decisions. Applying the model to music outside the Western Classical canon requires further research. Note that the Interpretation Map can be created using the categories not defined in this study. We have assumed a structural categorization and organization of interpretive decisions common to Western Classical music and a Classical piano trio. The model can be applied to other pieces and ensemble configurations with some adjustments.

The model was validated on signals measured during rehearsals. Cardiac response to music playing in other performance scenarios—individual practice, **auditions**, concerts **in major venue with large** audience—will likely differ (Mulcahy et al., 1990; Harmat and Theorell, 2009; Chanwimalueang et al., 2017). An extension of the model to account for various performance scenarios could add additional variables (fixed factors) related to specific conditions.

The results may not be representative of those of other populations, for example, non-professionals who may be grappling with learning issues, or cardiac patients who may have other physiological constraints. However, the methodology provides a framework translatable to studies with other groups.

The annotations for the Interpretation Map were made by the professional musicians themselves. The translatability of the presented framework to medical applications would require an evaluation of the robustness of the annotations made by professional musicians other than the performing trio and by non-professionals. Preliminary findings comparing musicians and non-musicians show that both groups select regions of interest similarly and identified common prominent bass notes, in a performance of Grieg's “Solveig's Song” and of Boulez's “Fragments d'une ébauche” (Bedoya Ramos, 2023). Future work will expand these findings to consider categories marked here by professional musicians.

We did not track other physiological signals, such as respiration, skin conductance, and movement. Adding these factors could help explain more of the variability in the RR intervals in future models. For example, tracking movement could help explain RR variability not directly related to the music (apart from random effects). However, the main goal of this study was to use only music features and to examine their effect on RR intervals relative to each other, which we found to be one of the model's strengths.

## 6 Conclusions

We have presented a novel framework for modeling the autonomic response in terms of the changes in RR intervals of musicians based on music features and the musicians' interpretation of the piece. Notably, by using only music information extracted from audio recordings or the music score, the models were able to explain about half of the variability of the RR interval series for all musicians (R^2^ = 0.540 for the pianist, 0.606 for the violinist and 0.494 for the cellist). We found loudness, climaxes, and moments of concern to be the most significant features. These features may be related to physical effort or mental challenges while performing the most demanding and engaging parts of a music piece. Another important feature was the starting factor, indicating the importance of separately modeling the initial physiological reaction to playing music.

To conclude, we have shown how instantaneous changes in RR intervals rely on time-varying expressive music properties and decisions and have created a framework for estimating performers' physiological reactions using music-based information alone. Active engagement in music-making is a fruitful area to explore for cardiovascular variability. Because listeners receive the results of these musical actions, the approach could also apply to modeling listeners' physiological responses to music and can be used to develop new non-pharmacological therapies. Future studies could be oriented toward comparing the autonomic response to music stimuli between healthy players and those with cardiovascular diseases. The analysis of differences between these groups of players would help in monitoring autonomic balance during music playing.

## Data Availability

The datasets presented in this article are not readily available due to privacy-related concerns. Requests to access the datasets should be directed to Prof. Elaine Chew (elaine.chew@kcl.ac.uk).

## Footnotes

^1^ http://mazurka.org.uk/cgi-bin/tapsnap
